# Risperidone in the treatment of conduct disorder in preschool children without intellectual disability

**DOI:** 10.1186/1753-2000-5-10

**Published:** 2011-04-13

**Authors:** Eyup S Ercan, Burge Kabukcu Basay, Omer Basay, Sibel Durak, Burcu Ozbaran

**Affiliations:** 1 Department of Child and Adolescent Psychiatry, Ege University School of Medicine, Izmir (35100), Turkey; 2 Department of Child and Adolescent Psychiatry, Dr. Behçet Uz Children's Hospital, Izmir (35210), Turkey

## Abstract

**Background:**

The DSM-IV-TR (Diagnostic and Statistical Manual of Mental Disorders, 4^th ^edition Textrevision) highlights the especially poor outcomes of early-onset conduct disorder (CD). The strong link between the patient's age at treatment and its efficacy points the importance of early intervention. Risperidone is one of the most commonly studied medications used to treat CD in children and adolescents. The aim of this study is to obtain preliminary data about the efficacy and tolerability of risperidone treatment in otherwise typically developing preschool children with conduct disorder and severe behavioral problems.

**Method:**

We recruited 12 otherwise normally developing preschoolers (ten boys and two girls) with CD for this study. We could not follow up with 4 children at control visits properly; thus, 8 children (six girls, two boys; mean age: 42.4 months) completed the study. We treated the patients with risperidone in an open-label fashion for 8 weeks, starting with a daily dosage of 0.125 mg/day or 0.25 mg/day depending on the patient's weight (<20 kg children: 0.125 mg/day; >20 kg children: 0.25 mg/day). Dosage titration and increments were performed at 2-week interval clinical assessments. The Turgay DSM-IV Based Disruptive Behavior Disorders Child and Adolescent Rating & Screening Scale (T-DSM-IV-S) as well as the Clinical Global Impression Scale (CGI) assessed treatment efficacy; the Extrapyramidal Symptom Rating Scale (ESRS) and laboratory evaluations assessed treatment safety.

**Results:**

The mean daily dosage of risperidone at the end of 8 weeks was 0.78 mg/day (SD: 0.39) with a maximum dosage of 1.50 mg/day. Based on the CGI global improvement item, we classified all patients as "responders" (very much or much improved). Risperidone was associated with a 78% reduction in the CGI Severity score. We also detected significant improvements on all of the subscales of the T-DSM-IV-S. Tolerability was good, and serious adverse effects were not observed. We detected statistically significant prolactin level increments (p < 0.05), but no clinical symptoms associated with prolactinemia.

**Conclusion:**

The results of this study suggest that risperidone may be an effective and well-tolerated atypical antipsychotic for the treatment of CD in otherwise normally developing preschool children. The findings of the study should be interpreted as preliminary data considering its small sample size and open-label methodology.

## Background

Conduct disorder (CD); the most severe type of disruptive behavior disorders (DBDs); is among the most common psychiatric disorders in childhood and adolescence. CD accounts for 30% to 50% of child and adolescent referrals in some clinics [1,2]. According to a recent survey using Diagnostic and Statistical Manual of Mental Disorders, 4^th ^edition Textrevision (DSM-IV-TR) criteria, the prevalence of CD combined with oppositional defiant disorder (ODD) is approximately 5% [3]. The DSM-IV-TR states that CD is characterized by a repetitive pattern of behavior that violates the rights of others or societal rules. The main four symptom categories of the disorder are: physical aggression or threats of harm to people or animals; destruction of property; acts of deceitfulness or theft; and serious violations of age-appropriate rules [4].

CD is a stable diagnosis over time and is associated with unfavorable outcomes. A 7-year longitudinal study of children with conduct disorder showed that less than 15% of the sample recovered by mid-to-late adolescence [5]. Other longitudinal studies have reported that 45% to 90% still met diagnosis criteria 3 to 4 years later [6]. According to other research reports, 40% of patients with CD are diagnosed with antisocial personality disorder as adults and may have a criminal record. Of those who do not, most manifest significant functional impairments in their relationships and at work [6]. These patients are also at great risk for developing substance use and mood, anxiety or somatoform disorders [7].

The DSM-IV divides CD into two categories according to the age of onset: childhood- and adolescent-onset. The DSM-IV-TR also highlights the poor outcomes for children whose behavior problems begin early in life. Youths with childhood-onset CD are more likely to exhibit persistent antisocial behaviors and higher rates of aggression. A prognosis of childhood-onset CD may be related to the impairment of academic and social performance during a period of mental and behavioral maturation [8].

There is no longer doubt that disruptive behaviors emerge in early childhood and exhibit moderate stability [9]. The appropriateness of applying DSM-IV diagnostic criteria for CD to preschool children, however, is an area of controversy [10]. Noncompliance and aggression are more common in early childhood compared to other developmental periods; thus, some authors have argued that atypical behaviors in preschool children may be transient developmental perturbations [11], whereas others have suggested that these behaviors may be a disturbance of the parent-child relationship [12]. Other concerns have centered on whether young children are developmentally capable of engaging in the behaviors that characterize the disorder and whether a functional equivalents of these behaviors exists across the time [10]. In their review of the evidence for the construct validity of DSM-based ODD and CD diagnoses, Keenan and Wakschlang (2002) emphasized that the determination of abnormality rests on establishing impairment in normal developmental functioning. A behavior's pervasiveness and intensity is critical to this determination [10]. The essential features of CD (i.e., violation of rules and the rights of others, aggressiveness and destructiveness) are applicable to preschool children because they are able to understand the concept of rules and can control their behavior accordingly [10,13]. To conclude, the authors stated that with some modification based on the child's developmental level, the DSM framework is a valid method for identifying preschool children with disruptive behaviors [10].

Kim-Cohen et al. labeled patients who met five or more CD criteria as having "moderate to severe" CD in their study for the validation of CD in 4.5-5 year-old children [14]. They found prevalence of the normal range of CD and "moderate to severe" CD among 4.5-5 year-old preschoolers as 6.6% and 2.5% respectively. In another study that compared referred and non-referred 2.5- to 5.5-year-olds, the prevalence of CD was 2% in the non-referred group and 41.8% in referred group. In the same study, the prevalence rates for ODD were 2% and 72.2% in the non-referred and referred group, respectively. DSM-IV symptoms of ODD and CD may distinguish referred from non-referred preschoolers in a pattern similar to that in older children. This possibility suggests that DSM-IV nosology is a valid diagnostic system for discriminating between typical and atypical disruptive behaviors in preschoolers [15].

The predictive validity of CD in preschoolers has also been documented. Preschoolers diagnosed with CD continue to have behavioral and educational problems 2 [14] and 5 years later [16] regardless of whether they have CD symptoms at follow up. In addition, the effect sizes between CD and its risk factors are comparable to those reported in older children [14,17]. Interventions to prevent chronic CD can be effective if applied early in life [18]. Kim, Cohen et al. (2005) showed that a minimum of DSM-IV-TR CD criteria were sensitive enough to identify preschoolers who might benefit from intervention [14].

Treating CD is important for several reasons. First of all, its symptoms may lead to severe difficulties in school life, social development and adult health, as mentioned above [19,20]; Second, the increased risk of physical injuries may be life-threatening for the patient and their victims. The large prevalence of CD and its adverse outcomes, the considerable stability continuity of diagnosis over time [1] and the risk of escalating aggressive and antisocial behavior in untreated patients [20] have directed many researchers to this topic in recent years. Treating CD should begin when the patient is diagnosed with CD because appropriate early interventions affect the disease's prognosis [18]. A review of multimodal treatment approaches for CD showed a strong link between the efficacy of treatment and the age of the patient at intervention, which demonstrates the value of early treatment [21]. Treatments should include multidisciplinary interventions due to the frequent co-occurrence of a number of biological, functional and psychosocial risk factors in the development of CD. Psychosocial therapies like behavioral therapy, psychotherapy and parental counseling and augmentation with pharmacotherapy are the most commonly used treatment modalities [22]. The presence of aggressive behavior in youths with a primary diagnosis of CD demonstrates the need for augmentation psychosocial therapy with pharmacotherapy [23].

Stimulants, typical and atypical antipsychotics and mood stabilizers are the medical agents used to treat CD and aggression. Most of these treatments have been shown to have some efficacy, although the expected side effect profile and decrease in symptom severity associated with each agent determines their differences. Among these, both typical and atypical antipsychotics can control the aggression and explosiveness of CD, but only typical antipsychotics are associated with the risk of extrapyramidal symptoms and tardive dyskinesia [24]. Mood stabilizers including lithium, carbamazapine and valproic acid have a variety of effectiveness in trials ranging from significant to minor decreases in symptom severity [8,25]. The need to closely monitor patients' blood drug level-especially in the case of lithium-has deterred its use in pediatric patients with CD. Stimulants (mostly methylphenidate) are also effective for controlling aggression, but these drugs are usually preferred in cases of CD comorbid with ADHD [26,27]. Two recently conducted reviews condensed the pharmacotherapy of CD and aggression [25,28]. In the first meta-analysis conducted by Ipser and Stein, lithium and the atypical antipsychotic risperidone were found to be effective in treating CD on both global measures and symptom severity. Risperidone was effective and demonstrated a safe side-effect profile. In the second review, Pappadopulos et al. reviewed all randomized controlled trials that were conducted with aggressive youths (i.e., age < 19). The diagnoses of the patients included mostly DBD, CD and ADHD, but other disorders with aggression as a core symptom were also included in this study. The atypical antipsychotic group, all of whom were prescribed with risperidone (nine trials), exerted a notably large overall Cohen's d effect size (mean ES = 0.9). Typical antipsychotics (two trials with haloperidole and one trial with thioridazine) exerted a medium ES (mean ES = 0.7). The ES for mood stabilizers (five trials with lithium and one trial with carbamazepine) was moderate (ES = 0.4); the variability in the efficacy of lithium was notable (ESs ranged between 0.0 and 0.9). Trials with stimulants (mostly methylphenidate over 20 trials) were conducted mostly with patients with a primary diagnosis of ADHD with comorbid DBD. Stimulants exerted a medium to large effect on pediatric aggression (mean ES = 0.78). Lastly, we reviewed four randomized controlled trials with atomoxetine and found the ESs to be small (mean ES = 0.18), implying that atomoxetine does not effectively control aggression.

Risperidone, which is an atypical antipsychotic with the potent properties of 5-HT2 and D2 receptor antagonists, is the most commonly studied agent in studies using the following designs: short-term double-blind, randomized and controlled in children and adolescents [7,19,24]; long-term and open-label [29-31]; and long-term, randomized, double-blind and placebo-controlled [32]. The results of these studies suggest that risperidone is an effective treatment of behavioral disturbances in children and adolescents with CD and that the benefits of this agent endure during maintenance treatments. Note that most of these studies were conducted using children with below average IQs.

A pooled analysis of the data obtained from two studies [7,19] on the effects of risperidone in children with below average IQs and DBD diagnoses showed that risperidone produced improvement in both social competence and problematic behavior (e.g., insecure/anxious and conduct problems), whereas affective insecurities (e.g., shy, timid, clings to adults, crying or tearful episodes) failed to improve [33].

Risperidone was also studied for its control of aggression in children and adolescents with bipolar disorder [34,35] and pervasive developmental disorder [36-39]. Again, risperidone was effective and safe in these trials and demonstrated a good side-effect profile, especially in low doses.

The treatment of CD in preschoolers lacks sufficient data and needs more care starting with diagnostic assessments. Proper developmental and functional assessment is crucial. Due to the lack of controlled trials and complete medication use evidence, psychotherapeutic interventions with the aim of increasing parenting skills or other therapy modalities such as behavioral therapy with parental involvement are recommended as first-line interventions in typically developing children. Carefully monitored medication is a second-step treatment for children with moderate to severe symptoms and functional impairments that persist after appropriate psychotherapeutic interventions [40]. Risperidone is recommended as the first medication choice for treating children with DBD with severe aggression without co-occurring ADHD [19,32,40,41].

In the few studies that have conducted risperidone trials in preschoolers, it was shown to be an effective and well-tolerated treatment. Furthermore, in an open-label 8-week trial using patients with bipolar disorder [42], an open-label 16-week trial using patients with pervasive developmental disorder [43], and a 6-month randomized placebo-controlled trial using patients with pervasive developmental disorder [39], risperidone was found to be beneficial and safe in low doses with no serious side effects in preschoolers; however, these trials reported significant gains in weight and prolactin levels. A 3-year follow-up study conducted in 53 preschoolers with pervasive developmental disorders revealed that risperidone effectively controls behavioral disorders and affect dysregulation in the long term [44]. Increased prolactin levels without clinical signs and an increased appetite were the most frequent side effects. One retrospective study of children with aggression associated with various diagnoses described a mean decrease of 36% in the severity of symptoms after using risperidone [45].

Developmentally inappropriate and severe behavioral disturbances are present in preschoolers [9]. These disturbances may severely interfere with relationships, social functioning and the development of the child. The DSM-IV criteria for conduct disorder are valid for this age group. Treating these children is crucial considering the remarkable stability of their symptoms, the risk for a future comorbid diagnosis and the efficacy of early interventions. Pharmacotherapy is needed when psychotherapic interventions are not successful. Some of our data suggest that risperidone treatments control aggression in this age group, but more evidence of this treatment's efficacy and tolerability in typically developing children with conduct disorder is needed.

In this open-label trial, we aimed to obtain preliminary data about the tolerability and efficacy of risperidone monotherapy in otherwise normally developing preschoolers with CD and severe behavioral problems. Furthermore, we sought to construct the baseline data for an extension study. To our knowledge, this study is the first to evaluate risperidone monotherapy in the treatment of preschoolers with both CD and normal intelligences.

## Method

We designed this study as an 8-week, open-label, single center trial. The Ethics Committee of the University approved the study procedure, and the study was conducted in accordance with the principles of the Declaration of Helsinki. All participants' parents gave written informed consent prior to the treatment.

All of the study procedures were completed at the Ege University School of Medicine Department of Child and Adolescent Psychiatry. This study center is a central medical school and tertiary referral hospital located in Izmir (population approximately 3 million), in Turkey. We determined participants from the patients referred to the study center from the two main state hospitals of Izmir as "treatment resistant and severely disturbed". These state hospitals have a low socioeconomic catchment area. Two child and adolescent psychiatry specialists evaluated and treated the patients at these hospitals' outpatient clinics. Patients who were diagnosed with conduct disorder and ADHD during their outpatient policlinic visits were recruited for the present study. Prior to referral, all patients had psychotherapeutic interventions to some extent, including parent training. Moreover, they were treated with a psychostimulant medication (i.e., short-acting methylphenidate). All participants were defined as "treatment resistant," having received minimal or no benefit at all from the previous treatment. No participants were on medication at the time of referral to our study center.

The senior author, an experienced child and adolescent psychiatry specialist, first assessed all children in the outpatient clinic of the university at recruitment, followed by two child and adolescent psychiatry residents. The senior author first interviewed children and then applied the Turkish version of the Kiddie-SADS Lifetime Version (K-SADS-PL) [46,47]. Although the K-SADS-PL is a semi-structured interview for use with school-age children, the K-SADS-E (Epidemiologic version) has previously been used to ascertain specific diagnoses, including ODD and CD in preschoolers [15]. In this study conducted by Keenan and Wakschlag some modifications to the K-SADS-E were made for the purpose of providing developmentally appropriate operational definitions. For example, to qualify as having stolen something, they did not require the item to be of non-trivial value as stated in DSM-IV because preschoolers do not usually attempt to take expensive items. Weapon use included sticks, rocks or bats. The participant age group in this study was very similar to our study group (mean age: 48 months versus 42 months, respectively) [15]. Another study applied the DSM-IV symptoms of CD to preschoolers; these symptoms included fighting, bullying, lying, stealing, behaving cruelly toward people or animals, vandalizing and violating rules [14]. The "forced sexual activity" item was excluded because it was deemed inappropriate for this age group [14]. Birmaher et al. (2009) completed a psychometric study to assess the reliability of the K-SADS-PL in preschoolers and suggested that the K-SADS-PL is a reliable tool to evaluate DSM-IV psychiatric disorders in preschoolers [48]. The use of the K-SADS in preschool may reduce method variance when trying to establish continuity and discontinuity between conditions in preschool and later in childhood [49].

In our study, we also modified "weapon use" (i.e., stone, stick, bat) and "stealing" (including trivial belongings) in line with Keenan and Wakschlag (2004) [15]. In actuality, most of the weapon-brandishing children used knifes with the intention of threatening others. Fighting, bullying, lying, behaving cruelly toward people or animals, destroying property and violating rules were behaviors present in most of the participants. (The rule violation criterion is considered to be present if the child knowingly breaks the rules at home or elsewhere. Most of the children's parents stated that they did not accept guests to their homes or felt unable to shop at markets with their children because they do not behave in age-appropriate ways.) Stealing (without confrontation) and fire setting were present in three children. Two children were reported to have run away from home many times during the day despite their parents' prohibition of this behavior. Forced sexual activity or stealing with confrontation, were not present in any of the children. (Note that preschool education is not obligatory in Turkey, and none of the children attended school. All of the parents reported that they could not send their children to school because of the behavioral problems; thus, truancy could not be evaluated.)

We diagnosed 16 children (13 boys 3 girls) with CD. All children had a comorbid ADHD diagnosis. Other psychiatric disorders that might have been present with temper tantrums or aggression (e.g., affective disorders) were ruled out following the K-SADS-PL. That same day, a comprehensive interview was performed with the parents. Furthermore, we took a complete medical, developmental and psychiatric history from the children. All of the children were from middle or low socioeconomic class families. Poor parenting, abuse, and negative family experiences contribute to the development of CD [50]; however, other than a low socioeconomic level, we did not detect any additional risk factors (e.g., abuse, neglect, physical violence, or parent rejection). Two mothers were taking antidepressants. Observations of the children in both a playroom and during the psychiatric assessment supported the psychiatric history in terms of aggressive behaviors and developmental characteristics. The children's medical history revealed that all of the children were severely aggressive toward their parents and other children. They bit, beat, kicked, scratched, threatened, swore at people, threw objects or stones at them, and frequently initiated fights, and some were violent toward animals. They lied, played with matches, used knifes, and broke and harmed furniture and belongings. Table 1 shows some of the children's demonstrative behavioral problems.

**Table 1:** Patient sociodemographic characteristics and behavioral problems

*No*	*Age (month)*	*Sex*	*Socioeconomic Status*	*Severe Behavioral Problems*
*1*	30	F	Low	Has punched her eardrum with matchstick, has drunk chemical cleaning agent, bits and kicks her peers, throws objects to people around.
*2*	44	F	Middle	Has started fire at home. Plays with knife, and is offensive. Her mother tells that she has been taking depression treatment due to the stress she has been living with her girl.
*3*	51	M	Middle	Has jumped from the balcony being "Batman". He usually lies, swears people and is very aggressive against his peers.
*4*	72	M	Low	Has been plucking his brother's hair, which has resulted in a localized alopecia. He kicks everybody at home. The family cannot accept guests to home due to this boy's offensive behaviors.
*5*	44	M	Low	Kicks his peers, plays with knife and runs after people with a knife in his hand, and tells that he's going to kill them.
*6*	38	M	Low	Climbs on to TV and jumps over, once almost fall under it. Throws objects to people, swears.
*7*	31	M	Low	Kicks and bites his parents at home, usually lies and is aggressive even against to older children. He is a famous child around.
*8*	29	M	Low	Plays with knife and matches. Spites and bites peers. His mother told that last time he pick up a knife from the kitchen and run after his grandfather shouting "I'll kill you"!

F:Female, M:Male

Parents of participants were offered a chance to take part in a parent-training program developed by Ercan and Aydın (1999) [51]. This program was based on Barkley's (1997) work and attempted to educate parents and other members of the family about disruptive behavior disorders and provide them with effective behavior-management techniques [52]. The program consisted of eight meetings (four consecutive weekly meetings, followed by four monthly meetings). Parents of four children stated that they would not be able to attend the meetings due to financial problems; thus, we excluded these children from the study. Parents of remaining 12 children attended the meetings. Eight parents attended more than 75% of the program; the other four attended more than 50%. No parents reported receiving a remarkable benefit at the end of the program. Socioeconomical problems might have contributed to the insufficient attendance. Furthermore, disadvantaged sociocultural characteristics might have prevented the parents from gaining the expected benefits of the program. At this point in the study, medications were offered to the parents for their children. We informed parents about the study; subsequently, they agreed to participate. The study began after the parents signed a written informed consent. All of the children were outpatients, and none were using medication at the beginning of the study.

The study began with 12 children (ten boys, two girls). At the first visit, a pediatrician neurology specialist performed a complete physical and neurologic examination; EEG and ECGs were taken, and blood biochemistry, a complete blood count and prolactin levels were evaluated to rule out any neurological and medical illnesses that could create a medication contraindication. None of the children demonstrated any laboratory assessment abnormalities in the EEG, ECG or physical or neurologic examinations.

To identify a developmental delay, an experienced psychologist administered the Ankara Developmental Screening Inventory (ADSI) on all participants. The ADSI consists of 154 items and measured the general development of the child using the sum of its four subscales: language/cognitive, fine motor, gross motor and social capability/self-attention. The inventory is obtained using the responses of the mother or the primary caregiver of the child on each item. The ADSI was developed using items from similar inventories from other countries, and its validity and reliability have been demonstrated in Turkish children [53]. One of the subscales assesses the child's cognitive level and provides data if there are any developmental delays in intellectual ability. Children with normal IQs were defined as those without developmental delays in general or normal cognitive development levels at the end of the assessment. None of the participants were diagnosed with a developmental disability, but two children had a minimal speech disability. Because other developmental properties were normal for their age group, we did not exclude them from the study.

We defined the inclusion criteria as follows.

1. Participants must be within the preschool age range. (In Turkey, the preschool age group is defined as 3-6 year-olds.)

2. Participants must be diagnosed with conduct disorder (comorbid ADHD diagnosis is allowed; other psychiatric diagnosis are not allowed).

3. Participants must be severely ill (CGI Scale score 6 or higher).

4. Participants must fail to respond to the parent-training program.

5. Participants must not be medicated at the time of entry (although all children had recently used a short-term short-acting methylphenidate).

6. Participants must be otherwise developmentally normal.

7. Participants cannot have an abnormality on any medical examination.

8. Parents must sign the written informed consent.

We treated all patients with risperidone starting with a daily dose of either 0.125 mg/day or 0.25 mg/day, depending on the weight of the child (<20 kg and ≥20 kg, respectively). The dosage amounts and medication schedule was based on Findling et al. (2000), who used a double-blind method to treat children with CD [24]. We made efficacy and side effect assessments in the study center at 2-week intervals with the aim of receiving more information and preventing unwanted events. Parents were called for brief telephone interviews each week. Medications were regulated individually; twice a week, an incremental dosage titration was made until we observed optimal therapeutic effects. We planned not to exceed a maximum 2-mg/day dosage. This maximum dosage was based on Biederman et al. (2004), who conducted an open-label 8-week trial using preschoolers with bipolar disorder [34]. We conducted final assessments at the end of Week 8. Psychotropic drugs other than risperidone were not allowed during the study.

Four children did not follow up the control visits properly. Their parents were called and briefly interviewed about the effects and side effects of the medication over the telephone. Parents did not report insufficient responses or adverse events due to the medication; however, because safety and efficacy assessments were not obtained regularly, we excluded these children from the study. All other participants (six boys and two girls) completed the study. Table 1 shows participant sociodemographic characteristics.

### Efficacy and Side Effect Assessments

#### 1. Efficacy assessments

Two scales assessed disease severity and improvement. 1) The clinician completed both the Severity (CGI-S; 1 = not ill, 7 = severely ill) and Improvement subscales (CGI-I; 1 = much improved, 7 = much worse) of the Clinical Global Impression Scale [54]. Lower scores reflect a reduced psychopathology and greater therapeutic effectiveness. The CGI-S was filled out at the beginning, during Week 4 and at the end (Week 8) of the study. The CGI-I was filled out during Weeks 4 and 8.

2) The Turgay DSM-IV Based Child and Adolescent Behavior Disorders Screening and Rating Scale, clinician and parent forms (T-DSM-IV-S-C & T-DSM-IV-S-P) assesses inattention (IA; nine items), hyperactivity-impulsivity (HI; nine items), opposition-defiance (OD; eight items) and conduct disorder (CD; 15 items) on a 4-point Likert-type scale (0 = not at all, 1 = just a little, 2 = quite a bit, and 3 = very much). Turgay et al. developed this scale, and Ercan et al. translated and adapted it for Turkish [55,56]. The T-DSM-IV-S is based on the DSM-IV diagnostic criteria. Greater scores reflect increases in symptom severity. The clinician and parents completed their respective forms of the scales at the beginning of the study, during Week 4 and at the end of the study.

The senior author, who also adapted and translated the T-DSM-IV-S into Turkish, scored each efficacy scale. "Response to medication" was defined as a 30% reduction in symptoms according to the T-DSM-IV-S or as having been judged as at least much improved on the CGI-I (i.e., ≤2).

#### 2. Side-effect assessments

1) Laboratory tests: Complete blood counts (CBC), blood biochemistry (including liver and kidney function tests, electrolytes and fasting glucose level), and prolactin levels were measured at the beginning and end of the study.

2) ECGs were recorded at the beginning and at the end of the study.

3) Child body weight was measured to assess weight change at the beginning of the study and at 2-week intervals.

4) The Extrapyramidal Symptom Rating Scale [57] assessed extrapyramidal adverse events. The clinician completed this scale at 2-week intervals, beginning during the second week of the study. In addition, a neurologic examination was performed at each visit to detect whether extrapyramidal side effects were present.

5) For capturing adverse effects the authors developed a checklist to evaluate probable adverse effects reported in the previous literature. The last item asks for any other side effect that is not present on the checklist. Symptom severity is scored on 4-point Likert scale (0 = not at all, 1 = mild, 2 = moderate, 3 = severe). The clinician assessed the patient at each visit by asking the items on the checklist one by one.

### Analytical Procedures

Statistical analyses were conducted using SPSS 13.0. Statistical analyses comprised paired-sample t-tests and one-way repeated-measures analysis of variance (ANOVAs). We adapted the LSD correction for pairwise comparisons. We considered P < 0.05 to be statistically significant.

## Results

The participant mean age was 42.4 (SD = 14.3) months. Two children were female, and the others were male. At the end of 8 weeks, the mean risperidone dose was 0.78 mg (SD = 0.39). The maximum dose was 1.50 mg/day, which was achieved in only one child. Risperidone doses used in this study were low; five children (62.5%) received less than 1 mg/day, two children (25%) received 1 mg/day and one child (12.5%) received 1.5 mg/day.

### Efficacy

**Figure 1:**
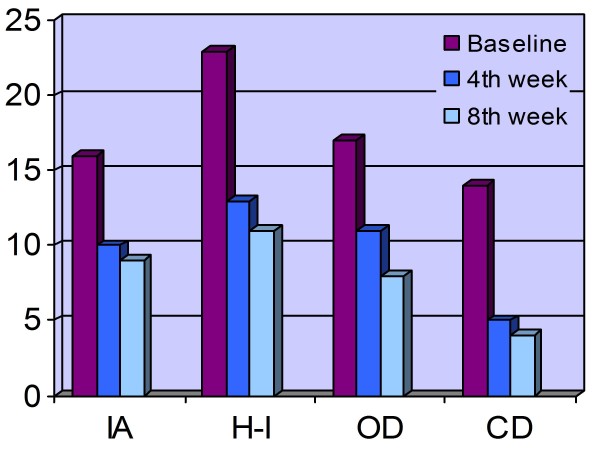
**T-DSM-IV-Parent Mean Scores**. IA: Inattention, H-I: Hyperactivity-Impulsivity, OD: Opposition defiance, CD: Conduct disorder

**Figure 2:**
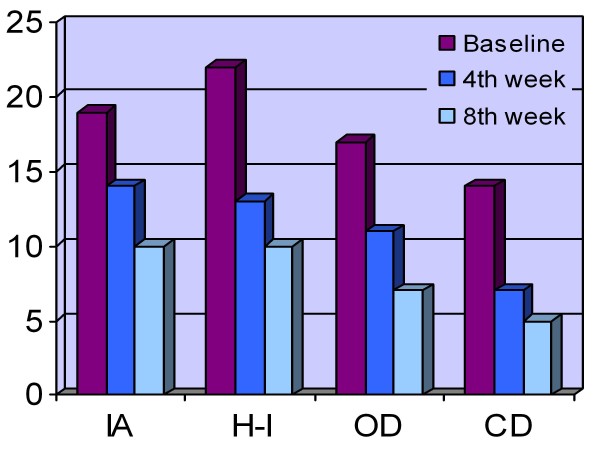
**T-DSM-IV-Clinician Mean Scores**. IA: Inattention, H-I: Hyperactivity-Impulsivity, OD: Opposition defiance, CD: Conduct disorder

After 8 weeks, we observed clinically significant improvements in all patients. The mean CGI-S score was 6.4 (SD = 0.5) at the beginning of the study (all participants were severely ill, scoring 6 or 7 on the CGI-S). At the end of the study, the mean CGI-S score was 1.4 (SD = 0.5). Risperidone was associated with a 78% reduction in the CGI-S score (p < 0.001). All of the children had greatly improved (CGI-I scores of 1 or 2). According to the T-DSM-IV-S, there was a total score reductions of 37.8 and 40.8 on the parental and clinical forms, respectively (SD = 19.2, p = 0.001; SD = 15.3, p < 0.001). All of the patients were classified as "responders" according to both the CGI and T-DSM-IV (parent and clinician) scales. We found statistically significant improvements on all subscales of the clinical and parental forms of the T-DSM-IV-S. Figures 1 and 2 as well as Table 2 show the mean scores of these scales with their comparisons at baseline as well as Weeks 4 and 8.

**Table 2:** Comparison of mean (SD) scores at baseline, Week 4 and Week 8

*Scale*	*Subscale*	*Rater*	Baseline* Mean(SD)	***4***^***th***^***week Mean(SD)***	***8***^***th***^***week Mean(SD)***	*F*	*Pairwise comparisons*
T-DSM-IV-S	IA	P	16.1(6.3)	9.6(4.1)	8.5(3.6)	6.129	8^th ^< Baseline(p = 0.021)
		C	18.9(5.1)	13.9(1.8)	10.1(2.1)	22.561	4^th ^< Baseline(p = 0.08) 8^th ^< Baseline(p = 0.01) 8^th ^< 4^th^(p = 0.01)
T-DSM-IV-S	H-I	P	22.6(4.5)	13.0(5.4)	11.0(3.3)	17.452	4^th ^< Baseline(p = 0.07) 8^th ^< Baseline(p < 0.001)
		C	21.6(2.8)	12.8(4.2)	9.6(2.8)	23.098	4^th ^< Baseline(p = 0.004) 8^th ^< Baseline(p < 0.001)
T-DSM-IV-S	OD	P	16.9(5.4)	10.9(6.2)	8.3(2.9)	11.818	4^th ^< Baseline(p = 0.039) 8^th ^< Baseline(p < 0.001)
		C	17.4(2.8)	11.4(2.8)	7.1(1.4)	33.126	4^th ^< Baseline(p = 0.001) 8^th ^< Baseline(p < 0.001) 8^th ^< 4^th^(p = 0.017)
T-DSM-IV-S	CD	P	14.0(8.6)	5.1(4.9)	4.0(2.1)	8.924	4^th ^< Baseline(p = 0.033) 8^th ^< Baseline(p = 0.007)
		C	14.0(4.8)	7.4(2.9)	4.5(2.4)	16.119	4^th ^< Baseline(p = 0.012) 8^th ^< Baseline(p = 0.001)
T-DSM-IV-S	Total	P	69.5(19.1)	38.9(19.1)	31.8(8.8)	13.393	4^th ^< Baseline(p = 0.020) 8^th ^< Baseline(p = 0.001)
		C	71.9(12.7)	46.9(11.1)	31.0(5.6)	30.410	4^th ^< Baseline(p = 0.003) 8^th ^< Baseline(p < 0.001) 8^th ^< 4^th^(p = 0.015)
CGI	Severity	C	6.4(0.5)	1.8(1.0)	1.4(0.5)	165.444	4^th ^&8^th ^< Baseline (p < 0.001)
CGI	Improvement	C	2.5(0.5)	1.8(0.7)	1.4(0.5)	6.785	4^th ^> Baseline(p = 0.048) 8^th ^> Baseline(p = 0.002)

T-DSM-IV-S: Turgay DSM-IV Based Child and Adolescent Behavior Disorders Screening and Rating Scale; CGI: Clinical Global Impression Scale; IA: Inattention; H-I: Hyperactivity-Impulsivity; OD: Opposition-Defiance; CD: Conduct Disorder; P: Parent; C: Clinician

*For CGI-Improvement Subscale assessments, 2^nd ^week was accepted as baseline

### Tolerability

All of the children tolerated the medication well. Parents of two children reported a mild to moderate sedation that disappeared after two weeks. The sedation was not present at the clinical visit, and neurologic examinations were normal. Sedation occurred after the first medication, and sleepiness occurred during the daytime. Sedation symptoms improved after 3 to 4 days and disappeared in a week. Sedation was not observed in the other children.

We did not observe a significant or clinically relevant weight gain in participants. Only one child gained 5.1% of their weight; all of the others gained less than 5%. The mean weight gain (±SD) from baseline was 0.3 ± 0.3 kg (p = 0.061; mean weight at baseline was 16.0 ± 3.4 kg)

Liver and kidney function, fasting blood glucose levels, blood electrolytes, complete blood count measurements and ECG recordings did not change over the duration of the study.

We detected a statistically significant, seven fold increase in prolactin levels at the end of the study. The mean change of prolactin was 33.9 ± 23.5 ng/ml (p < 0.05). The baseline mean prolactin level was 5.3 ± 1.4 ng/ml, and at Week 8, the mean prolactin level was 70.0 ± 21.9 ng/ml. Six children (one girl, five boys) had prolactin levels above the upper normal limit, but no participant showed clinical signs of hyperprolactinemia.

Nausea, vomiting, sedation and acute dystonic reaction developed in a patient who mistakenly received a high dose of risperidone (the patient's mother gave him a dose of 2.5 mg, instead of 0.25 mg, 0.01 mg/kg). The child's mother explained that after her child received the medication, the boy said he was feeling pain mostly in his neck and that upper extremity movements were difficult. The child and his mother were referred to the nearest hospital, and an intramuscular medication (biperidene) was given to the child. The pain, movement difficulty and muscle hardness were resolved in half an hour; however, the sedation and nausea lasted for nearly one day. Vomiting also occurred on the same day. The child visited our clinic the next day; his neurologic examination was normal, and extrapyramidal symptoms were absent. The patient was not started on oral biperidene because there were no symptoms. The risperidone treatment was stopped for a week, and the patient was monitored closely. No symptoms reoccurred. Risperidone tolerance was good after restarting the treatment one week later. None of the other children presented neurological side effects or extrapyramidal symptoms.

## Discussion

This study was a prospective open-label trial of risperidone to treat severe behavioral problems in preschoolers with CD who were otherwise developmentally normal. All 8 children (100%) were considered responders after 8 weeks of the trial.

We observed a gradual decline in scores on both the parent (P) and clinician (C) scales on all four symptom areas of the Turgay DSM-IV Based Child and Adolescent Behavior Disorders scale (inattention, hyperactivity-impulsivity, opposition defiance and conduct problems). Furthermore, we obtained significant differences between baseline and Week 4 comparisons as well as between baseline and Week 8 comparisons on the HI-P&C, OD-P, CD-P&C and CGI-S&I subscales. The IA-C and OD-C subscale comparisons revealed significant differences between baseline and Week 4 plus Week 8 as well as between Week 4 and 8. A statistically significant difference was present between baseline and Week 8 on the IA-P subscale. These results show that by Week 4, both parents and the clinician reported significant improvements on all four symptom areas and in global disease severity (with the exception of the IA-P subscale) after risperidone was administered. We interpret this result to mean that the beneficial effects of risperidone appear within one month. This finding is important when we consider that these children are at great risk to self-injure and have severely disturbed relationships. We do not think the benefits of this treatment are related to the sedation that may occur as a side effect. Sedation occurred only in two patients and disappeared after two weeks. Previous studies have also stated in that risperidone is an effective treatment for aggression and that improvement is not related to sedation [7,24].

Our results were consistent with previous studies that have assessed risperidone to treat disruptive behavior problems. Some of these studies used short-term double-blind methods; others used randomized controlled trials with child and adolescent patients [7,19,24]. The results of these studies suggest that risperidone is an effective agent to control the aggression and destructive behavior of children and adolescents with conduct disorder. In addition, long-term extension studies were conducted in either a open-label fashion [29-31] or a randomized, double-blind, placebo-controlled design [32]; again, risperidone was safe and effective when used as a maintenance treatment. Most of these studies were conducted with children and adolescents with below average IQs.

In a retrospective study of eight aggressive preschoolers without pervasive development disorder and comorbid diagnoses of attention deficit hyperactivity, bipolar, intermittent explosive and anxiety disorders, a risperidone treatment from 1 to 10 months in combination with therapy was effective in controlling aggression [45]. This study reported an 88% response rate and a 36% reduction in the CGI-Severity score. Significant weight gains were an adverse event of the treatment. Our study is similar to this previous study in terms of the age and symptoms of the patients; however, the study design, comorbid diagnosis and combination of other treatments are important differences. Our study found a more impressive response rate (100%) and a reduction in the CGI-Severity score (78%). The large difference in response may be related to the exclusion of children with comorbid diagnoses from our study.

Pandina et al. reviewed pilot studies, large clinical trials, and long-term open-label studies with more than 800 patients diagnosed with DBD [20]. Risperidone doses of 0.02 to 0.06 mg/kg/day were associated with target symptom improvement compared to placebo within one to four weeks of treatment. The most common side effects reported in the reviewed studies were somnolence, weight gain, headache, rhinitis, vomiting, dyspepsia and an increase in prolactin. Prolactin levels rapidly increased during the first month of treatment and then declined over the following year. The incidence of extrapyramidal side effects was low. Although sedation was reported frequently in these studies, significant drops in verbal learning or attention were not found. Moreover, cognitive functioning improved on several measures.

Unexpectedly, our study did not find any statistically significant weight gains in patients after 2 months (participant mean weight = 16 kg at baseline; mean weight = 16.3 kg at Week 8). Furthermore, parents did not report increased appetites in their children. This finding is not consistent with previous studies showing that weight gain occurred in several of the double-blind, placebo-controlled trials [20]. Note, however, that our study was short term, and weight gain might have been observed at a long-term follow up. Weight gain should be carefully monitored to prevent metabolic syndrome or Type-2 diabetes.

In addition to weight gain, some adverse effects such as flu-like symptoms and mild gastrointestinal symptoms were frequently reported in previous studies. We did not observe these side effects in our patients (excluding the nausea and vomiting that occurred in the child with acute dystonia). We did not use a specific test to assess patient cognitive function; however, at the clinical follow-up visit, parents were asked but did not report any cognitive impairment. Only two children had symptoms of sedation at the beginning of the treatment. Some parents stated that after treatment, their child maintained better focus on daily activities and play and constructed better social relationships. We found a significant decrease on the inattention subscale score of the T-DSM-IV-S, meaning that patients' attention improved. This finding is consistent with another risperidone trial that was conducted using youths with CD [58]. However, Findling et al. did not find that risperidone was associated with improvement in attention [24]; thus, this result should be interpreted cautiously.

The seven fold increase in prolactin was an expected but important outcome of risperidone treatment. Clinical disturbances did not accompany this increase. Elevated prolactin levels are one of the most commonly reported outcomes of risperidone treatment in previous studies. In some of the previous studies, these elevations were related to clinical adverse events such as gynecomastia, transient amenorrhea, or galactorrhea [29,31] and in some others were not related [7,19]. Pandina et al. stated in their review that hyperprolactinemia associated with risperidone treatment may be a transitory phenomenon of limited clinical significance; furthermore, they emphasized the need for additional studies [20]. In a study conducted with 25 children with autism between 3.9 and 7 years old, hyperprolactinemia due to risperidone treatment was not significantly correlated with age, weight, dosage amount or clinical outcomes [59]. Dunbar et al. found that the prolactin net area under curve (AUC) was not associated with a deviation from expected growth [60]. Moreover, they did not find a statistically significant correlation between prolactin levels and sexual maturation during risperidone treatment. The potential adverse effects of hyperprolactinemia, such as growth and maturation, must be carefully observed and considered when treating patients who experience prolacin elevation. Furthermore, future studies should investigate the effects of risperidone-related prolactin increases in young populations.

Other than prolactin levels, there were no laboratory test abnormalities (i.e., blood biochemistry, complete blood count or ECG). A short-term risperidone treatment conducted with 120 children and adolescents between the ages of 3 and 17 reported a non-significant alkaline phosphatase elevation in 52.5% of patients and a marked liver enzyme elevation in 0.8% of patients [61]. The authors stated that concomitant medication use was allowed in the study.

### Study Limitations

Few studies have examined the psychopharmacologic treatment of severely aggressive preschoolers with conduct disorder and otherwise normal developments. This study helps construct a baseline for future studies; however, highlighting the important limitations of this study is necessary. First of all, we planned to start this study with 16 participants, but four patients withdrew at the first meeting. Another four patients dropped out because they could not attend follow up visits. This small sample size (eight patients) clearly limits the statistical power of our analyses. Related to the statistical analyses, we did not perform multiple comparisons or control for other variables. Thus, we could not provide a specific estimate of the statistical power used to compute sample size, so the results should be considered as hypothesis generating rather than confirmatory. Another limitation is the shortage of assessment tools designed for preschoolers. The T-DSM-IV scale is based on the DSM-IV DBD diagnostic criteria, and although these criteria are valid in preschoolers, age-appropriate adaptations are needed. The short duration of this study is other important limitation. Although we did not detect patient weight gain, the risks of lipid metabolism related to antipsychotics are well documented. Furthermore, we do not know much about the long-term effects of prolactin increase in preschoolers. 8-week study duration is a short time to determine the safety of this procedure; long-term trials are necessary.

## Conclusion

The validity of conduct disorder has been recently well-established in preschoolers. Early intervention decreases the progress of the disease. Psychotherapy modalities such as parent training and behavioral therapies including the parents are first-line treatment modalities. However, for those cases who fail to respond to first-line treatments, clinicians face the challenge of the lack of empirical data on the efficacy and side effects of medications for young children. Risperidone is the most studied agent in youths to treat conduct disorder, but most of these data come from school-age children. Studies with preschoolers are usually conducted with developmentally delayed children. We believe, however, that treating severe behavioral problems in normally developing preschoolers needs investigation because adverse drug effects and drug efficacy may differ from older children. This preliminary open-label 8-week study suggests that a low dose of risperidone may be an effective treatment for conduct problems in preschoolers with an otherwise normal development and average intelligence. This drug was well tolerated with a small dosage titration and close monitoring. The results of this study should be interpreted as preliminary hypothesis-generating data because of its important limitations. Note once more that medications should only be considered in preschoolers if other behavioral treatment modalities and family interventions fail to provide benefits and the disorder obviously disturbs the development of the child by affecting their social relationships as well as their bodily and mental health. If the medication is started, it should be used cautiously with frequent follow-up interviews and close monitoring. Additional short-term and long-term randomized placebo-controlled trials are needed to evaluate the safety and efficacy of risperidone treatment in preschoolers.

## Competing interests

The authors declare that they have no competing interests.

## Authors' contributions

ESE conceived of the study, participated in its design, coordination and supervision as well as patient interviews and assessments. BKB participated in patient assessments, follow up procedures, sequence alignment and drafted the manuscript. OB participated in patient assessments, follow up procedures and performed the statistical analysis. SD took part in patient selection. BO participated in the coordination of the study. All authors read and approved the final manuscript.

## References

[B1] Steiner H Practice parameters for the assessment and treatment of children and adolescents with conduct disorder AACAP J Am Acad Child Adolesc Psychiatry 1997 36 Suppl 10 122 139 10.1097/00004583-199710001-00008 9334568

[B2] Kazdin A Treatment of Antisocial behavior in Children and Adolescents 1985 Homewood, Illinois; Dorsey Press

[B3] Joseph MR Walter G Soutullo CA Martin A, Volkmar FR Oppositional Defiant and Conduct Disorders Lewis's Child and Adolescent Psychiatry: Comprehensive Textbook 2007 4 Philadelphia: Lippincott Williams and Wilkins

[B4] American Psychiatric Association Diagnostic and Statistical Manual of Mental Disorders, DSM-IV-TR 2000 4 Washington, DC: American Psychiatric Association

[B5] Lahey BB Loeber R Burke J Rathouz PJ Adolescent outcomes of childhood conduct disorder among clinic-referred boys: Predictors of improvement J Abnorm Child Psychol 2002 30 4 333 348 10.1023/A:1015761723226 12108765

[B6] Christopher RT Sadock B, Sadok VA Disruptive Behavior Disorders Kaplan and Saddock's Comprehensive Textbook of Psychiatry 2005 8 Philadelphia: Lippincott Williams and Wilkins

[B7] Snyder R Turgay A Aman M Binder C Fisman S Carroll A The Risperidone Conduct Study Group: Effects of risperidone on conduct and disruptive behavior disorders in children with subaverage IQ's J Am Acad Child Adolesc Psychiatry 2002 41 9 1026 1036 10.1097/00004583-200209000-00002 12218423

[B8] Burke D Loeber R Birmaher B Oppositional defiant disorder and conduct disorder: A review of the past 10 years, Part II J Am Acad Child Adolesc Psychiatry 2002 41 11 1275 1293 10.1097/00004583-200211000-00009 12410070

[B9] Wakschlag LS Leventhal BL Thomas JM with commentary by Pine D Regier D, First M, Narrow W Disruptive Behavior Disorders & ADHD in Preschool Children: Characterizing Heterotypic Continuities for a Developmentally-Informed Nosology for DSM-V Age and Gender Considerations in Psychiatric Diagnosis: A Research Agenda for DSM-V Washington DC: American Psychiatric Publishing Inc in press

[B10] Keenan K Wakschlag LS Can a Valid Diagnosis of Disruptive Behavior Disorder Be Made in Preschool Children? Am J Psychiatry 2002 159 351 358 10.1176/appi.ajp.159.3.351 11869995

[B11] Campbell SB Behavior Problems in Preschool Children: Clinical and Developmental Issues New York, Guilford 2002

[B12] Anders T Sameroff A, Emde R Clinical syndromes, relationship disturbances and their assessment, in Relationship Disturbances in Early Childhood: A Developmental Approach 1989 New York, Basic Books 25 144

[B13] Kochanska G Murray KT Coy KC Inhibitory control as a contributor to conscience in childhood: from toddler to early school age Child Dev 1997 68 263 277 10.2307/1131849 9180001

[B14] Kim-Cohen J Arseneault L Caspi A Tomas PM Taylor A Moffitt TE Validity of DSM-IV conduct disorder in 4 1/2-5-year-old children: a longitudinal epidemiological study Am J Psychiatry 2005 162 1108 1117 10.1176/appi.ajp.162.6.1108 15930059

[B15] Keenan K Wakschlag LS Are Oppositional Defiant and Conduct Disorder Symptoms Normative Behaviors in Preschoolers? A Comparison of Referred and Nonreferred Children Am J Psychiatry 2004 161 2 356 358 10.1176/appi.ajp.161.2.356 14754786

[B16] Kim-Cohen J Arseneault PL Newcom R Adams F Bolton H Cant L Delgado K Freeman J Golaszewski A Kelesidi K Matthew K Mountain N Oxley D Watson S Werts H Caspi A Moffitt TE Five-year predictive validity of DSM-IV conduct disorder research diagnosis in 41/2-5-year-old children Eur Child Adolesc Psychiatry 2009 18 284 29 10.1007/s00787-008-0729-1 19165535 PMC4212821

[B17] Rowe R Maughan B Pickles A Costello EJ Angold A The relationship between DSM-IV oppositional defiant disorder and conduct disorder: findings from the Great Smoky Mountains Study J Child Psychol Psychiatry 2002 43 365 373 10.1111/1469-7610.00027 11944878

[B18] Olds DL Henderson CR Cole R Eckenrode J Kitzman H Luckey D Pettitt L Sidora K Morris P Powers J Long-term effects of nurse home visitation on children's criminal and antisocial behavior: 15-year follow-up of a randomized controlled trial JAMA 1998 280 1238 1244 10.1001/jama.280.14.1238 9786373

[B19] Aman MG De Smedt G Derivan A Lyons B Findling RL Risperidone Disruptive Behavior Study Group: Double-blind, placebo-controlled study of risperidone for the treatment of disruptive behaviors in children with subaverage intelligence Am J Psychiatry 2002 159 1337 1346 10.1176/appi.ajp.159.8.1337 12153826

[B20] Pandina G Aman M Findling R Risperidone in the management of disruptive behavior disorders J Child Adolesc Psychopharmacol 2006 16 4 379 392 10.1089/cap.2006.16.379 16958564

[B21] Moretti MM Emmrys C Grizenko N Holland R Moore K Shamsie J Hamilton H The treatment of conduct disorder: Perspectives from across Canada Can J Psychiatry 1997 42 637 648 9288427 10.1177/070674379704200611

[B22] Kazdin AE Parent management training: Evidence, outcomes and issues J Am Acad Child Adolesc Psychiatry 1997 39 414 420 10.1097/00004583-200004000-00009 9334547

[B23] Kutcher S Aman M Brooks SJ Buitelaar J van Daalen E Fegert J Findling RL Fisman S Greenhill LL Huss M Kusumakar V Pine D Taylor E Tyano S International consensus statement on attentin deficit/hyperactivity disorder (ADHD) and disruptive behavior disorders (DBDs): Clinical implications and treatment practice suggestions Eur Neuropsychopharmacol 2004 14 11 28 10.1016/S0924-977X(03)00045-2 14659983

[B24] Findling RL McNamara NK Branicky LA Schluchter MD Lemon E Blumjer JL A double blind pilot study of risperidone in the treatment of conduct disorder J Am Acad Child Adolesc Psychiatry 2000 39 4 509 516 10.1097/00004583-200004000-00021 10761354

[B25] Ipser J Stein DJ Systemic review of pharmacotherapy of disruptive behavior disorders in children and adolescents Psychopharmacology 2007 191 127 140 10.1007/s00213-006-0537-6 16983542

[B26] Greenhill LL Pliszka S Dulcan MK Bernet W Arnold V Beitchman J Practice parameters for the use of stimulant medications in the treatment of children, adolescents, and adults AACAP J Am Acad Child Adolesc Psychiatry 2002 41 2 Suppl 26 49 10.1097/00004583-200202001-00003 11833633

[B27] Günther T Herpertz-Dahlmann B Jolles J Konrad K The influence of risperidone on attentional functions in children and adolescents with attention-deficit/hyperactivity disorder and co-morbid disruptive behavior disorder J Child Adolesc Psychopharmacol 2006 16 6 725 735 17201616 10.1089/cap.2006.16.725

[B28] Pappadopulos E Woolston S Chait A Perkins M Connor DF Jensen PS Pharmacotherapy of agression in children and adolescents: Efficacy and effect size J Can Acad Child Adolesc Psychiatry 2006 15 1 27 39 18392193 PMC2277275

[B29] Turgay A Binder C Synder R Fisman S Long-term safety and efficacy of risperdone for the treatment of disruptive behavior disorders in children with subaverage IQs Pediatrics 2002 110 e34 10.1542/peds.110.3.e34 12205284

[B30] Findling RL Aman MG Eerdekens M Derivan A Lyons B the Risperidone Disruptive Behavior Study Group Long-term, open-label study of risperidone in children with severe disruptive behavior disorders and below-average IQ Am J Psychiatry 2004 161 677 684 10.1176/appi.ajp.161.4.677 15056514

[B31] Croonenberghs J Fegert JM Findling RL De Smedt G Van Dongen S Risperidone Disruptive Behavior Study Group: Risperidone in children with disruptive behavior disorders and subaverage intelligence: A 1-year, open-label study of 504 patients J Am Acad Child Adolesc Psychiatry 2005 44 64 72 10.1097/01.chi.0000145805.24274.09 15608545

[B32] Reyes M Buitelaar J Toren P Augustyns I Eerdekens M A randomized, double-blind, placebo-controlled study of risperidone maintenance treatment in children and adolescents with disruptive behavior disorders Am J Psychiatry 2006 163 402 410 10.1176/appi.ajp.163.3.402 16513860

[B33] Aman M Buitelaar J De Smedt G Wapenaar R Binder C Pharmacotherapy of disruptive behavior and item changes on a standardized rating scale: Pooled analysis of risperidone effects in children with subaverage IQ J Child Adolesc Psychopharmacol 2005 15 2 220 232 10.1089/cap.2005.15.220 15910206

[B34] Biederman J Mick E Wozniak J Aleardi M Spencer T Faraone SV An open-label trial of risperidone in children and adolescents with bipolar disorder J Child Adolesc Psychopharmacol 2005 15 2 311 317 10.1089/cap.2005.15.311 15910215

[B35] Frazier JA Meyer MC Biederman J Wozniak J Wilens TE Spencet TJ Kim GS Shapiro S Risperidone treatment for juvenile bipolar disorder: A retrospective chart review J Am Acad Child Adolesc Psychiatry 1999 38 960 965 10.1097/00004583-199908000-00011 10434487

[B36] Research Units of Pediatric Psychopharmacology (RUPP) Autism Network Risperidone in children with autism and serious behavioral problems N Engl J Med 2002 347 314 321 10.1056/NEJMoa013171 12151468

[B37] Research Units of Pediatric Psychopharmacology (RUPP) Autism Network Risperidone treatment of autistic disorder: Longer-term benefits and blinded discontinuation after six months Am J Psychiatry 2005 162 1361 69 10.1176/appi.ajp.162.7.1361 15994720

[B38] Shea S Turgay A Carroll A Schulz M Orlik H Smith I Dunbar F Risperidone in the treatment of disruptive behavioral symptoms in children with autistic and other pervasive developmental disorders Pediatrics 2004 114 5 e634 641 10.1542/peds.2003-0264-F 15492353

[B39] Luby J Mrakotsky C Stalets MM Belden A Heffelfinger A Williams M Spitznagel E Risperdione in preschool children with autistic spectrum disorders: An investigation of safety and efficacy J Child Adolesc Psychopaharmacology 2006 16 5 575 587 10.1089/cap.2006.16.575 17069546

[B40] Gleason MM Psychopharmacological Treatment for Very Young Children: Contexts and Guidelines J Am Acad Child Adolesc Psychiatry 2007 46 12 1532 1572 10.1097/chi.0b013e3181570d9e 18030077

[B41] Connor DF Glatt SJ Lopez I Psychopharmacology and aggression: A meta-analysis of stimulant effects on overt/covert aggression-related behaviors in ADHD J Am Acad Child Adolesc Psychiatry 2002 41 253 261 10.1097/00004583-200203000-00004 11886019

[B42] Biederman J Mick E Hammerness P Harpold T Aleardi M Dougherty M Wozniak J Open-label, 8-week trial of olanzapine and risperidone for the treatment of bipolar disorder in preschool-age children Biol Psychiatry 2005 58 589 594 10.1016/j.biopsych.2005.03.019 16239162

[B43] Masi G Cosenza A Mucci M De Vito G Risperidone monotherapy in preschool children with pervasive developmental disorders J Child Neurol 2001 16 6 395 400 11417603 10.1177/088307380101600602

[B44] Masi G Cosenza A Mucci M Brovedani P A 3-year naturalistic study of 53 preschool children with pervasive developmental disorders treated with risperidone J Clin Psychiatry 2003 64 9 1039 1047 10.4088/JCP.v64n0909 14628979

[B45] Cesena M Gonzalez-Heydrich J Szigethy E Kohlenberg TM DeMaso DR Case Report: A case series of eight aggressive young children treated with risperidone J Child Adolesc Psychopharmacol 2002 12 4 337 345 10.1089/104454602762599880 12625994

[B46] Kaufman J Birmaher B Brent D Rao U Flynn C Moreci P Williamson D Ryan N Schedule for Affective Disorders and Schizophrenia for School-Age Children-Present and Lifetime Version (K-SADS-PL): Initial reliability and validity data J Am Acad Child Adolesc Psychiatry 1997 36 980 988 10.1097/00004583-199707000-00021 9204677

[B47] Gokler B Unal F Pehlivanturk B Cengel Kultur E Akdemir D Taner Y Reliability and valıdıty of Schedule for Affective Disorders and Schizophrenia for School-age Children-Present and Lifetime Version-Turkish Version (K-SADS-PL-T) [in Turkish] Cocuk ve Genclik Ruh Saglığı Dergisi (Turkish Journal of Child and Adolescent Mental Health) 2004 11 3

[B48] Birmaher B Schedule for affective disorders and schizophrenia for school-age children (K-SADS-PL) for the assessment of preschool children - A preliminary psychometric study J Psychiatr Res 2009 43 7 680 686 10.1016/j.jpsychires.2008.10.003 19000625 PMC2736874

[B49] Egger HL Erkanli A Keeler G Potts E Walter BK Angold A Test-retest reliability of the preschool age psychiatric assessment (PAPA) J Am Acad Child Adolesc Psychiatry 2006 45 5 538 49 10.1097/01.chi.0000205705.71194.b8 16601400

[B50] Holmes S Slaughter JR Kashani J Risk Factors in Childhood That Lead to the Development of Conduct Disorder and Antisocial Personality Disorder Child Psychiatry Hum Dev 2001 31 3 10.1023/A:1026425304480 11196010

[B51] Ercan ES Aydın C Dikkat Eksikligi Hiperaktivite Bozuklugu. Istanbul, Gendas, Yayınevi 1999 *[Attention Deficit Hyperactivity Disorder, Istanbul, Gendas Publications]*

[B52] Barkley RA Managing the Defiant Child: A Guide to Parent Training 1997 New York, Guilford Press

[B53] Savasır I Sezgin N Erol N Ankara Developmental Screening Inventory [in Turkish] 1993 2 Ankara, Turk Psikologlar Dernegi (Turkish Psychological Association)

[B54] National Institute of Mental Health Clinic Global Impressions Scale (CGI) Psychopharmacol Bull 21 839 843

[B55] Turgay A Disruptive Behavior Disorders Child and Adolescent Screening and Rating Scales for Children, Adolescents, Parents and Teachers West Bloomfield (Michigan), Integrative Therapy Institute Publication 1994

[B56] Ercan ES Amado S Somer O Çıkoğlu S Development of a test battery for the assessment of attention deficit hyperactivity disorder [in Turkish] Cocuk ve Genclik Ruh Saglığı Dergisi (Turkish Journal of Child and Adolescent Mental Health) 2001 8 132 144

[B57] Chouinard G Ross-Chouinard A Annable L Jones BD The extrapyramidal symptom rating scale Can J Neurol Sci 1980 7 233

[B58] Ercan ES Kutlu A Cıkoglu S Veznedaroglu B Erermis S Varan A Risperidone in children and adolescents with conduct disorder: Asingle-center, open-label study Curr Ther Res Clin Exp 2003 64 1 55 64 10.1016/S0011-393X(03)00006-7 PMC4053023 24944356

[B59] Masi G Cosenza A Mucci M Prolactin levels in young children with pervasive developmental disorders during risperidone treatment J Child Adolesc Psychopharmacol 2001 11 4 389 394 10.1089/104454601317261564 11838821

[B60] Dunbar F Kusumakar V Daneman D Schulz M Growth and sexual maturation during long term treatment with risperidone Am J Psychiatry 2004 161 918 920 10.1176/appi.ajp.161.5.918 15121661

[B61] Erdogan A Atasoy N Akkurt H Ozturk D Karaahmet E Yalug I Yalug K Ankarali H Balciolu I Risperidone and liver function tests in children and adolescents: A short-term prospective study Prog Neuropychopharmacol Biol Psychiatry 2008 32 3 849 57 10.1016/j.pnpbp.2007.12.032 18258348

